# Preliminary Study: The Effectiveness of Nutrition Education Intervention Targeting Short-Statured Pregnant Women to Prevent Gestational Stunting

**DOI:** 10.3390/nu15194305

**Published:** 2023-10-09

**Authors:** Zuriati Muhamad, Trias Mahmudiono, Chrysoprase Thasya Abihail, Nur Sahila, Martina Puspa Wangi, Bagong Suyanto, Nurul Ashikin Binti Abdullah

**Affiliations:** 1 Public Health Doctoral Program, Faculty of Public Health, Universitas Airlangga, Surabaya 60115, Indonesia; zuriatimuhamad85@gmail.com; 2 Midwifery Department, Faculty of Health Science, Universitas Muhammadiyah Gorontalo, Gorontalo 96181, Indonesia; 3 Center for Health and Nutrition Education, Counseling, and Empowerment (CHeNECE), Department of Nutrition, Faculty of Public Health, Universitas Airlangga, Surabaya 60115, Indonesia; 4 Department of Nutrition, Faculty of Public Health, Universitas Airlangga, Surabaya 60115, Indonesia; abihailthasya@gmail.com (C.T.A.); martina.puspa.wangi-2019@fkm.unair.ac.id (M.P.W.); 5 Department of Health Policy and Administration, Faculty of Public Health, Universitas Airlangga, Surabaya 60115, Indonesia; nur.sahila-2018@fkm.unair.ac.id; 6 Department of Sociology, Faculty of Social and Political Sciences, Universitas Airlangga, Surabaya 60115, Indonesia; bagong.suyanto@fisip.unair.ac.id; 7 Institute of Biological Sciences, Faculty of Science, Universiti Malaya, Kuala Lumpur 50603, Malaysia; shikin84@um.edu.my

**Keywords:** short pregnant women, gestational stunting, cadres, child, health well-being

## Abstract

A short mother with a height < 150 cm is likely to give birth to a short baby with a body length < 48 cm so that later this short baby will become stunted. The success rate of stunting malnutrition prevention and control with specific methods is 30% and the success rate with sensitive methods is 70%. The size at risk for short pregnant women is equal to 30.5%. A major effort to improve the health status of short pregnant women and prevent stunting is to empower short pregnant women with the help of health professionals. This study aimed to analyze the effectiveness of providing nutrition education to pregnant women who have short stature to prevent gestational stunting. This study used a quantitative approach with a quasi-experimental design in the intervention group and the control group. Research results showed that there are differences in the knowledge, attitudes, and actions of pregnant women about prenatal care services before and after the intervention, as well as knowledge of pregnant women about nutrition before and after intervention. The support of the cadres had a great influence on the intervention group compared with the control group, which received only one module. Pregnant women’s knowledge of nutritional diets and pregnant women’s knowledge of antenatal care (ANC) services directly influence the delivery timing. Interventions to improve the health status of short pregnant women and prevent stunting neonates can be improved by improving the knowledge, attitudes, and behavior of short pregnant women about antenatal care, and knowledge of pregnant women about nutritional intake. The Short Pregnancy Medical Framework Support Model was developed for use in providing support to short pregnant women to prevent infant stunting.

## 1. Introduction

Pregnant women with short stature who have a height of 150 cm and a body mass index (BMI) < 18.5 kg significantly give birth to short babies < 48 cm and babies with a low birth weight (LBW) < 2500 g [1]. This happens between generations because the growth of the fetus in the womb is hampered because pregnant women are malnourished and experience a lack of vitamins and minerals in the body, causing a low birth weight (LBW) and stunting in newborns; this often occurs in low- and middle-income countries [2].

The problem of stunting that occurs in toddlers is caused by the baby’s body length at birth < 48 cm (neonate stunting) and, generally, babies born with a low birth weight (LBW), namely < 2500 g [3]. Stunted neonates are born from short mothers who have a height < 150 cm. The mother’s height affects the linearity of the offspring during the growth period on birth weight and length, including the mother’s nutritional intake during pregnancy [4]. Research conducted by Schmidt in West Java concluded that for every 1 cm increase in a pregnant women’s height, the baby’s body length will increase by 0.196 cm (*p* < 0.000). This is the important reason why women should be the important target in stunting improvement until the next generation [5].

Stunting is a condition of failure to thrive in infants (0–11 months) and toddlers (12–59 months) due to chronic malnutrition, especially in the first 1000 days of life, resulting in children too short for their age. Malnutrition occurs from infancy in the womb and during childhood early after the baby is born, but stunting conditions only appear after the child is 2 years old [6]. Stunting is one of the major problems in developing countries. It is estimated that the prevalence of stunting in children less than 5 years old in African and Asian countries (excluding China) is 90%, representing around 125 million individuals [7]. The global target that has been achieved was to reduce stunting from 39.7% in 1990 to 26.7% in 2010. Within 20 years, this can be reduced by 1.6% per year. A very small decrease occurred in Africa from 40% to 38%. Meanwhile, a significant decrease occurred in Asia (49% to 28%), namely, a decrease of around 2.9% per year [7].

The determinant factors of stunting in infants are the mother’s height < 150 cm and body mass index (BMI) < 18.5 cm, the mother’s weight gain is below standard, and her nutrient intake is below the nutritional adequacy level [7]. Research conducted in Sweden showed that short pregnant women were associated with a progressive increase in women giving birth to premature babies. Maternal short stature is a contributing factor to idiopathic preterm birth worldwide, possibly due to maternal anatomical constraints. Preterm birth is not only the leading cause of neonatal death worldwide but is also associated with adverse short-term and long-term health outcomes [8].

Based on the 2018 Basic Health Research (Riskesdas) data in Indonesia, height is a risk factor for pregnant women, namely < 150 cm, by 30.5%. There are as many as 34.2% short pregnant women in rural areas and 27.6% in urban areas. From the Riskesdas data, the higher the education level of pregnant women, the lower the prevalence of height as a risk < 150 cm. Chronic Energy Deficiency (CED) in pregnant women aged 15–49 years is 17.3% [9]. Research in Uruguay shows that short stature, with TB mothers < 160 cm, is a significant predictor of giving birth to stunted children [10]. These results are in line with previous studies in Brazil which looked at the relationship between short maternal stature and a low birth weight (<3000 g; *p* = 0.01) and stunting (*p* = 0.019) [11].

Health promotion, including nutrition education, is very influential in increasing a person’s knowledge, including pregnant women, about health information. Research conducted in Aceh shows that nutrition education interventions are effective in increasing knowledge about stunting prevention behavior in pregnant women (*p* = 0.002) [12]. In addition, providing interactive nutrition education has proven effective in increasing knowledge, attitudes, and actions related to nutrition and health in groups of pregnant women who receive interventions [13]. Based on the explanation above, researchers are interested in researching the effectiveness of providing nutrition education to pregnant women who have short stature to prevent gestational stunting.

## 2. Materials and Methods

This study was a quasi-experimental research design with pre-tests and post tests conducted on both the control group and the treatment group before and after the intervention was administered. The control group were provided with an intervention in the form of a leaflet about maternal health and nutrition, while the intervention group was provided with an intervention in the form of a leaflet along with healthcare worker accompaniment. The healthcare worker accompaniment was conducted to enhance understanding and monitoring processes for pregnant mothers with short stature.

**Figure 1:**
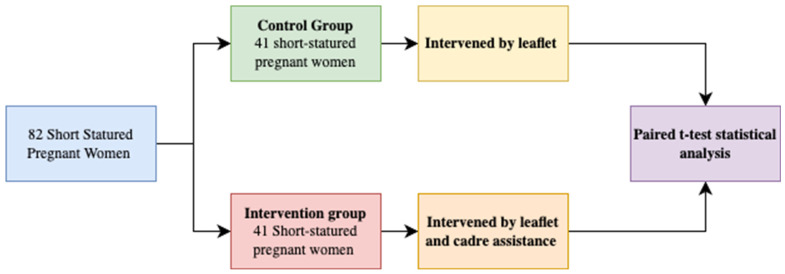
Stages of research and observation.

These three research stages are described in the following schema:

Figure 1 explains the stages of research and observation. The participants in this study were short pregnant mothers with a height of less than 150 cm, totaling 82 participants. The participants will be separated into two groups consisting of 41 participants for each group. The control group intervened with leaflets only and the intervention group intervened with leaflets and cadres’ assistance for 6 months. The stages of research and observation of this study can be seen further in Figure 1.

In order to execute the level 3 research which involves intervention activities, sample measurement was conducted to discover the number of the participants included in the research. We use the Cohen formula (1988) to calculate the number of participants for each group.

$$n=\frac{2x{\left(Z_{\frac{\alpha }{2}}+Z_{\beta }\right)}^{2}x\sigma^{2}}{\delta^{2}}\;=\frac{2x{\left(1.96+0.2\right)}^{2}x\sigma^{2}}{\delta^{2}}\;=\frac{2x{\left(1.96+0.2\right)}^{2}x5.06^{2}}{2.29^{2}}\;=40.96\text{\textasciitilde{}}41\mathrm{samples}$$

The total sample of the research was 41 respondents per group, representing the intervention group receiving cadres as an advisor and the control group, who only received modules. Inclusion criteria for the research sample were pregnant women of short stature with a height of less than 150 cm, residing in and being born in the research location, having a gestational age of 4–6 months from March to May 2021 (second trimester), having a history of contact with midwives and health cadres, excluding pregnant women with Chronic Energy Deficiency (CED), being literate, and willing to participate as research respondents from the beginning to the end of data collection.

The material provided to short pregnant women included in the leaflet media covered basic knowledge about Antenatal Care (ANC) services, the benefits of ANC, ANC service standards, and pregnancy risks. Pregnant women and health cadres have the opportunity to discuss health behaviors during pregnancy and recognize potential complications that may arise during the pregnancy period. Cadres were responsible for providing information about pregnancy care, the services available, signs of pregnancy dangers, and necessary actions to be taken.

The intervention of providing nutrition education and empowering pregnant women through cadre assistance was carried out for 9 months, starting from late March 2021 until November 2021. Univariate analysis was conducted to obtain descriptive insights into each research variable. The short pregnant women involved in the study, both in the control and intervention groups, underwent pre-tests and post tests based on a questionnaire formulated in accordance to the provided material. Prior to conducting assistance, the health cadres received training from the Nutrition Department of Tilango Health Center, with the head of the department working as a nutrition expert. The content covered during the training included nutritional intake during pregnancy and neonatal stunting prevention. The training method employed was roleplay, aimed at enabling pregnant women to use the educational materials provided effectively. Each roleplay session concluded with feedback and improvements provided by the speaker to enhance the quality of cadres’ assistance. The training was not a one-time event before its implementation; rather, it was carried out throughout the research period and the researcher observed and evaluated the cadres’ capacity to provide assistance.

The statistical analysis employed entailed the utilization of a paired *t*-test methodology, leveraging both pre-test and post test data. The purpose behind employing the paired *t*-test for each variable was rooted in the utilization of pre-test and post test data. This testing procedure was conducted across both the control and intervention groups, with the intent of ascertaining variations prior to and subsequent to the intervention within the intervention group. Conversely, the statistical assessment within the control group aimed to substantiate alterations transpiring in the absence of any form of intervention.

## 3. Results

### 3.1. Number of Pregnant Women and Cadres Included in The Research

#### 3.1.1. Pregnant Women Included in the Research

By applying the inclusion and exclusion criteria to the research sample, a total of 82 pregnant women were obtained and used as the research sample. These 82 pregnant women were divided into two groups: the intervention group and the control group. The intervention group received a nutritional education module accompanied by guidance from health cadres, while the control group received a nutritional education module without guidance from health cadres. The pregnant women involved in the study were divided into four treatment areas and four control areas.

**Figure 2:**
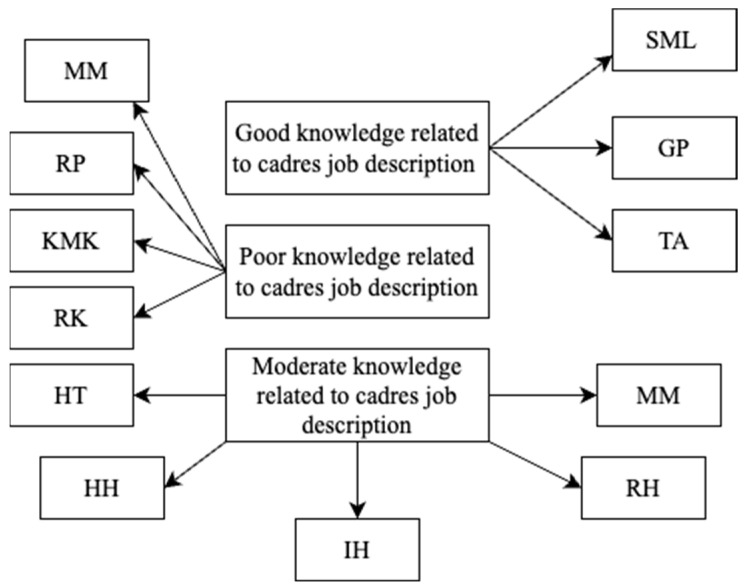
In-depth interview result with respondents related to stunted maternal knowledge of cadres’ job description.

Based on the qualitative information found in the research, it was obtained that the knowledge of pregnant mothers regarding the roles and functions of community health workers varies. There are two or three pregnant mothers who have good knowledge about the roles and functions of community health workers. There are four community health workers who have insufficient knowledge about their roles and functions, and there are five community health workers who have adequate knowledge about their roles and functions. The results of the in-depth-interview can be seen further on Figure 2.

Based on the information provided by short pregnant women as respondents, it was found that the age distribution of 20–35 years is the most dominant group in both the intervention and control groups. Table 1 explains the characteristics of the respondents interviewed based on the research groups.

The results of the study regarding the respondents’ characteristics revealed that the majority of respondents ages were within the range of 20–35 years, with a percentage of 68.3% in the intervention group and 73.2% in the control group. The educational levels of the respondents varied from basic education to higher education. The percentage of mothers with basic education in the intervention group was 24.4%, compared to 41% in the control group. The percentage of mothers with a high school education was 36.6% in the intervention group and 24% in the control group. The most dominant occupation was housewives, accounting for 87.7% in the control group and 65.9% in the intervention group. In terms of the frequency of Antenatal Care (ANC) visits, both the control and intervention groups underwent ANC examinations more than four times during the course of pregnancy. The number of parities in the control group was also predominantly less than four.

#### 3.1.2. Respondents Included in the Research

The individuals involved in the first stage of the study, which is qualitative research, are village midwives assigned to each area that serves as the research locus. According to the village midwives, community health workers (cadres) have been collaborating with midwives in assisting pregnant women. The assistance provided by the cadres to the village midwives during Antenatal Care (ANC) examinations includes measuring height, weight, and anthropometric criteria under the supervision of midwives.

Other respondents involved in this research are health cadres who assist short pregnant women. In addition to providing information about the schedule of integrated health post (posyandu) visits, the cadres’ tasks include providing services to pregnant women during posyandu sessions. This includes opening posyandu registrations, conducting anthropometric measurements, recording pregnancy examination results, providing education about nutritious food, delivering additional food to pregnant women, and monitoring pregnant women at home who have not undergone pregnancy examinations.

The cadres participating in the study receive guidance to ensure that the assistance provided not only enhances knowledge but also improves the attitudes and behaviors of short pregnant women in maintaining their health during pregnancy. The model of health cadres assistance for short pregnant women is developed based on the latest data from previous research, combined with input from relevant stakeholders.

Based on the results of the first stage of the study, the obtained propositions contribute to the formulation of an empowerment model for short pregnant women in preventing neonatal stunting. Propositions that contribute to the development of an empowerment model for short pregnant women to prevent neonatal stunting include the knowledge of short pregnant women about cadres, the access of short pregnant women to healthcare services, community support for short pregnant women, and the dietary habits or traditions of pregnant women during pregnancy. On the other hand, factors hindering the assistance of health cadres to short pregnant women include the pregnant women’s understanding of the role of health cadres and insufficient knowledge of pregnant women about nutritional intake during pregnancy.

### 3.2. Effect of Cadres Guidance towards Nutrition Knowledges and Behavior

After conducting Phase I research consisting of a qualitative study, followed by Phase II research involving model formulation, Phase III research tests the effectiveness of the intervention provided. Before carrying out the intervention, healthcare workers are provided with training to provide support to pregnant mothers using materials related to knowledge, attitudes, and actions towards pregnancy services, and nutritional intake. On the other hand, no support is given to the control group and there are no healthcare workers assigned to accompany pregnant mothers in the control group.

Table 2 demonstrates that the results of the *t*-test within the control and intervention groups reveal significant mean differences subsequent to the conducted study. This is substantiated by the one-tailed significance values of <0.05 across all variables. Within the study, no significant differences were observed, with two-tailed significance values > 0.05. This substantiates that the intervention involving the guidance of health aides for short-term pregnant women led to a significant enhancement, evidenced by significantly higher scores compared to the control group, which solely received modules.

Variables encompassing maternal knowledge, attitudes, pregnancy service practices, and nutritional intake exhibited substantial score improvements following guidance provided by health aides, as indicated by significance values < 0.05. Derived from the obtained outcomes, it can be concluded that significant score disparities are present within the intervention group, which received assistance from aides, across the four variables under scrutiny.

Based on Table 3, the results of the t-test between the control and intervention groups show that there is a significant difference in the mean values after the study. This is determined by the two-tailed *p*-value, which is <0.05 for all variables. At the beginning of the study, there were no significant differences, with a two-tailed *p*-value > 0.05. This demonstrates that the intervention involving healthcare worker accompaniment for short-statured pregnant mothers leads to a significant increase, which is notably higher compared to the control group that only received leaflets.

The variables of knowledge, attitudes, practices of pregnant mothers in pregnancy services, and nutritional intake experience a significant increase in scores after receiving accompaniment from healthcare workers, with significance values < 0.05. Based on the collected data, it can be concluded that there is a difference between before and after the intervention through healthcare worker accompaniment for these four variables.

In this multiple linear regression analysis, the backward analysis method was used for estimation to find the best approach for multiple independent variables. The backward method involves a stepwise elimination process where all X variables are regressed with the Y variable. The elimination of X variables is based on the smallest partial F value, and whether an X variable remains in the model is determined by the critical F value from the table. The backward method is a suitable regression approach as it explains the behavior of the response variable optimally by selecting explanatory variables from a pool of available data variables [14].

In the ANOVA regression table, the Sig. value of 0.000 indicates that the five X variables have a significant linear relationship with the birth length variable. For multiple linear regression, the equation is obtained by eliminating equations from the least squares method. The coefficients for knowledge, attitudes, actions in antenatal care, family support, and knowledge about nutritional intake can be obtained using Statistical Product and Social Science (SPSS) 25.0 software, following the table.

The effective estimations involve two variables that emerge in Model 4, namely the knowledge variable of pregnant mothers regarding antenatal care, with a coefficient of 0.109, and knowledge about nutritional intake, with a coefficient of 0.180. This can be interpreted as a tendency for an increase in birth length with the rise of values in these two variables. Effective estimation for birth length is achieved by considering knowledge about pregnancy care (ANC) and knowledge about nutritional intake. The coefficient of determination (R2) for the above regression equation using the backward method is 0.789. This value signifies that 78.9% of the variance in the birth length variable can be explained by the combined variances of the two X variables through the regression equation. In other words, around 21.1% of the variance in birth length is influenced by other variables not included in the analysis.

## 4. Discussion

The handling of stunting is carried out through specific interventions and sensitive interventions. Specific interventions refer to the target of the first 1000 days of life. Sensitive interventions are generally carried out outside the health sector. The target is the public. However, if sensitive interventions are explicitly planned and integrated with specific activities, the impact is sensitive to the safety of the growth and development process of the first 1000 days of life. The success rate of preventing and overcoming stunting problems through specific interventions is 30% and success rates through sensitive interventions are 70% [15,16].

One way to handle stunting through sensitive interventions is community empowerment. Community empowerment is an effort to increase the ability of the community to behave healthily, to be able to deal with health problems independently, play an active role in every health development, and be a driving force in realizing development with a health perspective. In this study, the main target in community empowerment was short pregnant women through the assistance of health cadres. Considering that previous studies have shown that the determinants of shortness in infants include the mother’s height being less than <150 cm [15].

Based on the results of the study, the assistance provided by the health cadres to the intervention group had a significant difference in the knowledge, attitudes, and actions of mothers in providing pregnancy services, and knowledge of pregnant women about nutritional intake. Different from the intervention group, the control group had no significant difference in the knowledge, attitudes, and actions of mothers in providing pregnancy services, and knowledge of pregnant women about nutritional intake. This is because in the intervention group all short pregnant women were accompanied by health cadres to provide information/education about pregnancy risk factors, danger signs of pregnancy, and antenatal care. On average, in each village, one to two women with short pregnancies are accompanied by one health cadre. Short pregnant women were accompanied from the second trimester when the study took place (±6 months of pregnancy until the time of delivery and measuring the length of the newborn’s body). Pregnant women as respondents participated in the provision of information/education quite enthusiastically. Health cadres will visit homes or meet pregnant women at posyandu every month. Pregnant women are becoming increasingly aware of the health of pregnant women during pregnancy and childbirth.

According to the WHO (World Health Organization) theory cited by Notoatmodjo (2007), one form of health object can be described by knowledge obtained from one’s own experience. Knowledge is a very important domain for the formation of one’s actions. From experience and research, it turns out that behavior based on knowledge will be more lasting than behavior that is not based on knowledge [1].

Based on the results of the study, the attitude of respondents increased significantly (*p* < 0.05). Respondents became positive after knowing that their opinion/attitude about pregnancy care was wrong and would have a big impact or risk on the health status of pregnant women. Health cadres are needed to facilitate health activities in the community. Health cadres who are active in the community will bring changes in behavior in the community.

It is in line with research conducted by Vika and Arulita (2015) in Rembang District, who found that there were differences in pre-test and post test scores for attitudes in the experimental group (*p* = 0.001 < 0.05) and the control group (0.001 < 0.05) in pregnant women after being given assistance by health cadres on the attitudes of pregnant women in carrying out ANC visits [17]. This is different from research conducted by Ketut Suarayasa (2017) in the city of Palu showing that there was no difference (*p* > 0.005) in the attitude of pregnant women in the treatment group and the control group before and after being given treatment in the form of assistance [18].

The results of the study showed that there were significant differences in the behavior of pregnant women before and after being given an intervention through the assistance of health cadres for antenatal care (*p* < 0.05). This is because the better the knowledge, the better the attitude will be. This good attitude will then be practiced by the individual in their daily behavior as well.

The results of the study are in line with research conducted by Rodiah et al. (2016), showing that there is a significant difference between the before and after of the intervention in the group that received empowerment from cadres compared to the group without empowerment from cadres [19].

That is in line with the study conducted by Brynne that cadres are an important tool in reducing maternal and child mortality. Evidence of the quality of the performance of cadres is seen from their performance in preventing malaria, providing health education to the community, promoting the benefits of exclusive breastfeeding for breastfeeding mothers, and the importance of caring for newborns. All the activities of these cadres showed good performance and results for the community and psychosocial support [20].

This research is a preliminary study that requires further research to confirm the validity of the research design and methods. The limitations of this research include the relatively uniform educational material provided to pregnant women, which raises the possibility that pregnant women may seek personal information, leading to an increase in their knowledge beyond the provided intervention. Furthermore, the results of the univariate analysis indicate that there is no diversity in terms of the age of the pregnant women involved in this study. Most of pregnant women were aged 20–35, classified as young adults, while pregnant women under the age of 20 constitute a minority group. Considering that chronic energy deficiency is more common in pregnant women under the age of 20, which is the background for the occurrence of gestational stunting, it cannot be confirmed whether the education provided can improve the knowledge of short pregnant women across all age groups. Despite the long duration of the study, there were no drop-outs. A limitation of this research is that the sample selection was conducted using purposive sampling, resulting in a smaller sample size compared to other intervention studies”.

## 5. Conclusions

Community empowerment is an effort to prevent stunting through a sensitive approach, where a sensitive approach has a higher success in reducing stunting compared to a specific treatment. Empowering short-term pregnant women through assisting health cadres is expected as cadres can assist pregnant women to increase their knowledge, understanding attitudes, behavior, and awareness to be able to access pregnancy services and be able to consume nutritious food that is useful for the health of the mother and fetus. If the mother has a healthy lifestyle, it will prevent gestational stunting from occurring. Assistance from health cadres can increase knowledge of the attitudes and actions of pregnant women in ANC services. This can be seen from the increasing number of visits to Posyandu by pregnant women every month. Knowledge of pregnant women about ANC services (pregnancy) has a significant effect on birth length. If the mother has good knowledge and understanding of the benefits of pregnancy services for the health of the mother and fetus, the mother will be diligent in having her pregnancy checked at the Posyandu; from the results of these examinations, the mother can find out the growth and development of the fetus every month and can minimize the risk of gestational stunting. Knowledge of pregnant women’s nutritional intake has a significant effect on birth length. If the mother has knowledge and understanding in choosing and processing nutritious food that is useful for pregnant women and fetal growth, it will minimize the occurrence of gestational stunting.

## Figures and Tables

**Table 1:** Respondent characteristics interviewed by group.

Characteristic		Group	
		Intervention	Control
		n (%)	n (%)
Age			
	<20 years old	9 (22.0)	2 (4.9)
	20–35 years old	28 (68.3)	30 (73.2)
	>35 years old	4 (9.8)	9 (22.0)
Education			
	Elementary	10 (24.4)	17 (41.0)
	Middle School	14 (34.1)	13 (32.0)
	Senior School	15 (36.6)	10 (24.0)
	Diploma	1 (2.4)	0 (0.0)
	Bachelor	1 (2.4)	1 (2.0)
Occupation			
	Housewives	36 (87.8)	27 (65.9)
	Freelance	2 (4.9)	11 (26.8)
	Student	1 (2.4)	2 (4.9)
	Civil Servant	1 (2.4)	1 (2.4)
	Farmer	1 (2.4)	0 (0.0)
ANC Frequency			
	Less than 4 times	0 (0.0)	1 (2.4)
	More than 4 times	40 (97.6)	41 (100.0)
Number of Parity			
	More than 4 times	37 (90.2)	37 (90.2)
	Less than 4 times	4 (9.8)	4 (9.8)

**Table 2:** Analysis of differences in knowledge, attitudes, and pregnancy care actions, and nutritional intake knowledge before and after the intervention given.

Variable	Mean ± SD		*p* Value (Paired *t* Test)
	Before	After	
Knowledge			
Intervention	29.85 ± 3.75	42.02 ± 3.73	<0.001 *
Control	30.05 ± 3.33	30.24 ± 3.20	0.088
Behavior			
Intervention	26.46 ± 3.20	41.59 ± 3.72	<0.001 *
Control	26.12 ± 2.57	26.32 ± 2.31	0.088
Practice			
Intervention	27.12 ± 2.97	36.44 ± 4.16	<0.001 *
Control	26.85 ± 2.79	27.39 ± 2.40	0.052
Nutrition Knowledge			
Intervention	28.98 ± 4.27	38.41 ± 5.06	<0.001 *
Control	28.76 ± 4.39	29.27 ± 3.47	0.231

*: Significant with Paired *t*-test using alpha 5%.

**Table 3:** Analysis of comparison between knowledge, attitudes, and actions of short stature pregnant mothers in pregnancy services, and knowledge about nutritional intake.

Variable	Mean ± SD		*p* Value (Paired *t* Test)
	Before	After	
Knowledge			
Intervention	29.85 ± 3.	30.05 ± 3.33	0.804
Control	42.02 ± 3.73	30.24 ± 3.20	<0.001 *
Behavior			
Intervention	26.46 ± 3.20	26.12 ± 2.57	0.596
Control	41.59 ± 3.72	26.32 ± 2.31	<0.001 *
Practice			
Intervention	27.12 ± 2.97	26.85 ± 2.79	0.674
Control	36.44 ± 4.16	27.39 ± 2.40	<0.001 *
Nutrition Intake			
Intervention	28.98 ± 4.27	28.76 ± 4.39	0.819
Control	38.41 ± 5.06	29.27 ± 3.47	<0.001 *

*: Significant with Paired t-test using alpha 5%.

## Data Availability

The data presented in this study are available on request from the corresponding author. The data are not publicly available due to the privacy of the respondents.
